# Enhancement of Osteoblastic-Like Cell Activity by Glow Discharge Plasma Surface Modified Hydroxyapatite/β-Tricalcium Phosphate Bone Substitute

**DOI:** 10.3390/ma10121347

**Published:** 2017-11-23

**Authors:** Eisner Salamanca, Yu-Hwa Pan, Aileen I. Tsai, Pei-Ying Lin, Ching-Kai Lin, Haw-Ming Huang, Nai-Chia Teng, Peter D. Wang, Wei-Jen Chang

**Affiliations:** 1 School of Dentistry, College of Oral Medicine, Taipei Medical University, Taipei 110, Taiwan; eisnergab@hotmail.com (E.S.); shalom.dc@msa.hinet.net (Y.-H.P.); payinglin53@gmail.com (P.-Y.L.); hhm@tmu.edu.tw (H.-M.H.); dianaten@tmu.edu.tw (N.-C.T.); 2 Department of Dentistry, Chang Gung Memorial Hospital, Taipei 105, Taiwan; ait001@adm.cgmh.org.tw (A.I.T.); philipcklin@msn.com (C.-K.L.); 3 Graduate Institute of Dental & Craniofacial Science, Chang Gung University, Taoyuan 333, Taiwan; 4 School of Dentistry, College of Medicine, China Medical University, Taichung 404, Taiwan; 5 Graduate Institute of Biomedical Materials & Tissue Engineering, College of Oral Medicine, Taipei Medical University, Taipei 110, Taiwan; 6 Dental Department, Taipei Medical University Hospital, Taipei 110, Taiwan; 7 Dental Department, Taipei Medical University, Shuang-Ho Hospital, Taipei 235, Taiwan

**Keywords:** HA/β-TCP, argon glow discharge plasma, guided bone regeneration, osteoconduction, cell viability, differentiation

## Abstract

Glow discharge plasma (GDP) treatments of biomaterials, such as hydroxyapatite/β-tricalcium phosphate (HA/β-TCP) composites, produce surfaces with fewer contaminants and may facilitate cell attachment and enhance bone regeneration. Thus, in this study we used argon glow discharge plasma (Ar-GDP) treatments to modify HA/β-TCP particle surfaces and investigated the physical and chemical properties of the resulting particles (HA/β-TCP + Ar-GDP). The HA/β-TCP particles were treated with GDP for 15 min in argon gas at room temperature under the following conditions: power: 80 W; frequency: 13.56 MHz; pressure: 100 mTorr. Scanning electron microscope (SEM) observations showed similar rough surfaces of HA/β-TCP + Ar-GDP HA/β-TCP particles, and energy dispersive spectrometry analyses showed that HA/β-TCP surfaces had more contaminants than HA/β-TCP + Ar-GDP surfaces. Ca/P mole ratios in HA/β-TCP and HA/β-TCP + Ar-GDP were 1.34 and 1.58, respectively. Both biomaterials presented maximal intensities of X-ray diffraction patterns at 27° with 600 a.u. At 25° and 40°, HA/β-TCP + Ar-GDP and HA/β-TCP particles had peaks of 200 a.u., which are similar to XRD intensities of human bone. In subsequent comparisons, MG-63 cell viability and differentiation into osteoblast-like cells were assessed on HA/β-TCP and HA/β-TCP + Ar-GDP surfaces, and Ar-GDP treatments led to improved cell growth and alkaline phosphatase activities. The present data indicate that GDP surface treatment modified HA/β-TCP surfaces by eliminating contaminants, and the resulting graft material enhanced bone regeneration.

## 1. Introduction

Multiple regenerative procedures have been developed for the treatment of deep infrabony defects, furcation involvements, and for socket preservation after tooth extraction [1,2,3,4]. Autogenous bone grafts remain the gold standard for bone regeneration procedures because they contain viable osteoblasts, organic and inorganic matrices, and biological modifiers. Although autogenous bone grafts are ideal for hard-tissue grafts, they are disadvantaged by limited availability, the tendency toward partial resorption, the requirement for additional surgery, and the ensuing increases in morbidity [5]. Thus, further studies are urgently required to develop and compare alternatives to allogenic grafts, including optional biomaterials such as xenogenic grafts from the same and other species, alloplastic materials, and synthetic and inorganic implant materials [2,6]. These materials may provide scaffolds for bone formation (osteoconduction) and could contain bone-forming cells (osteogenesis) or bone-inductive substances (osteoinduction) [3]. However, it remains unclear which graft materials are the most suitable for bone regeneration [7,8,9].

In 2005, Trombelli et al. showed that various forms of hydroxyapatite (HA) significantly improve clinical attachment levels compared with periodontal surgery [2]. Moreover, in 2003, Reynolds et al. reported good clinical outcomes following the use of calcium phosphate ceramic in periodontal therapy [6]. Subsequently, biphasic calcium phosphates (BCPs) comprising mixtures of HA and beta tricalcium phosphate (β-TCP) at varying ratios were well documented as bioinert and bioactive alloplastic materials [10,11]. In particular, the ratio of approximately 60% HA to 40% β-TCP produced optimal resorption by the material and maintenance of osteoconductive properties [12,13,14,15].

Glow discharge plasma (GDP) is formed by the passage of electric current through a low-pressure gas and is widely used for cleaning, sterilizing, and modifying biomaterial surfaces [16,17,18,19,20,21]. Glow-discharge techniques are well established cleaning methods in the microelectronics industry and are under current consideration in the production of biomaterials [22,23]. Glow-discharge methods offer great advantages with respect to the possible range of modifications. Using appropriate GDP parameters with argon, plasmas can remove all traces of potentially problematic entities from biomaterial surfaces, including contaminants, impurities, and native oxide layers [16], warranting further characterization of sputtered HA/β-TCP biomaterial surfaces after GDP treatment.

Bone scaffold can be manufactured by several synthetic routes developed to prepare BCP bioceramics of variable HA/β-TCP ratios simulating the physical and biological properties of natural bones [24]. Multiple techniques, such as gas foaming, freeze drying, thermally induced phase separation, precipitation, hydrolysis, mechanical mixture, among others [25]. While using these manufacturing techniques few papers have reported biomaterials sterilization processes, storage conditions, and the impurities that cover the materials [24]. Indeed, HA/β-TCP surface impurities removal using Argon plasma sputtering, has not been well documented and needs to be better understood.

Herein, a surface modification of HA/β-TCP particles using GDP treatments and an evaluation of enhancements in bone regeneration properties were carried out in the present study. Specifically, we determined the physical and chemical properties of HA/β-TCP particles after GDP surface treatment (HA/β-TCP + Ar-GDP) and tested the resulting biological effects on MG-63 cell viability and differentiation into osteoblast like cells.

## 2. Materials and Methods

### 2.1. Sample Preparation

Bone substitute granules of 500–1000 μm biphasic ceramic materials comprising 60% HA and 40% β-TCP (MBCP) were purchased from Biomatlante (Vigneux-de-Bretagne, France).

### 2.2. GDP Sputtering

**Figure 1:**
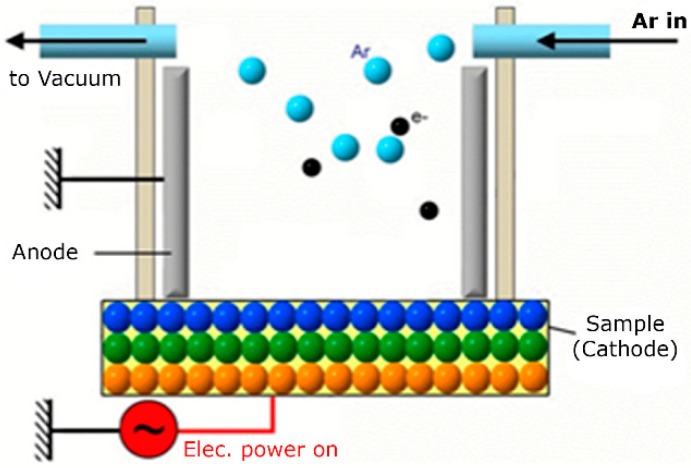
Glow discharge plasma design.

HA/β-TCP particles were treated for 15 min with GDP (PJ; AST Products Inc., North Billerica, MA, USA) in the presence of argon gas at room temperature under the following conditions: power: 80 W; frequency: 13.56 MHz; pressure: 100 mTorr (Figure 1).

### 2.3. Surface Topography Evaluations

Surface morphologies of HA/β-TCP + Ar-GDP were observed and compared with those of HA/β-TCP using 30 scanning electron microscopy images (SEM; Model 2400; Hitachi, Ltd., Tokyo, Japan) from the same sample. Prior to imaging, 25 nm thick layers of palladium gold were sputter-coated onto a sample using a sputtering apparatus (IB-2; Hitachi, Ltd., Tokyo, Japan). The evaluation was conducted with one sample only (*n* = 1).

### 2.4. Energy Dispersive Spectrometry

Elemental analyses of HA/β-TCP + Ar-GDP samples were performed using the same SEM apparatus described above coupled with an energy dispersive X-ray spectrometer (EDS; Model 2400; Hitachi, Ltd., Tokyo, Japan) (*n* = 1).

### 2.5. X-ray Photoelectron Spectroscopy (XPS)

Elemental and chemical analyses of treated surfaces were performed using X-ray photoelectron spectroscopy (ESCA system; VG Scientific, West Sussex, UK) with a 1486.6 eV monochromatic Al X-ray source. Spectra were collected at a normalized electron take-off angle to the sample surfaces. High resolution [C1s], [O1s], [Ca2p], and [P2p] spectra were obtained from 25 surface-treated samples (*n* = 1).

### 2.6. X-ray Diffraction Analyses

Crystalline structures and chemical compositions using approximately 0.5 mg of HA/β-TCP + Ar-GDP and HA/β-TCP, were analyzed using powder X-ray diffraction (XRD, X’Pert^3^ Powder, PANalytical Co. Ltd., Almelo, The Netherlands), at 40 kV and 40 mA with a scanning speed of 0.5°/s and a scanning range of 20°–50° (*n* = 1).

### 2.7. Cell Viability Assays

Cytotoxicity was assessed in vitro according to international standards for the evaluation of dental materials (ISO 7405:2008) Cytotoxicity was assessed in vitro according to international standards for the evaluation of dental materials (ISO 7405:2008) Cytotoxicity was assessed in vitro according to international standards for the evaluation of dental materials (ISO 7405:2008) Cytotoxicity was assessed in vitro according to international standards for the evaluation of dental materials (ISO 10993-5:2009 [26]). Passage 17 of the MG-63 osteoblast-like cells was seeded into 24-well Petri dishes (Nunclon; Nunc, Roskilde, Denmark) at a density of 1 × 10^4^ cells/mL using 1 mL of Dulbecco’s modified Eagle’s medium (DMEM; HyClone, Logan, UT, USA) supplemented with l-glutamine (4 mmol/L), 10% fetal bovine serum (FBS), and 1% penicillin streptomycin in every well for 24 h. The media was then removed and substituted with a new media consisting of the previous described DMEM + HA/β-TCP + Ar-GDP or HA/β-TCP particles in a concentration of 1 g/10 mL using just the media for the test wells. Control wells used the same DMEM media first described without any particle graft. Cells were cultured over periods of 1, 3, and 5 days at 5% CO_2_, 37 °C and 100% humidity. The 0 h starting point was defined as the moment when DMEM + particle grafts were added to the test wells.

At Days 1, 3, and 5, cell viabilities were assessed according to metabolic activities using (3-4,5-dimethylethiazol-2-yl)-2,5-diphenyl tetrazolium bromide (MTT) reduction assays. In these experiments, MTT was metabolically reduced by mitochondrial dehydrogenase in viable cells and the resulting production of colored formazan was determined according to the manufacturer’s instructions (MTT kit, Roche Applied Science, Mannheim, Germany). After removing MTT containing media from cells, formazan crystals were dissolved in 500 μL of dimethyl sulfoxide for 5 min, and optical density absorbance was recorded using an enzyme linked immunosorbent assay (ELISA) reader at 570 nm. Results were expressed as percentages (*n* = 6).

### 2.8. Cell Morphology

To investigate cell morphology on biomaterial particles, cells were cultured into 24-well Petri dishes (Nunclon; Nunc, Roskilde, Denmark) at a density of 1 × 10^4^ cells/mL osteogenic inductive media, which was a combination of the media described in the cell viability assay plus HA/β-TCP + Ar-GDP or HA/β-TCP particles in a concentration of 1 g/10 mL using 1 mL of media and particle grafts. Images were recorded at 40× magnification on Days 1, 3, and 5 using an optical microscope (Olympus BH-2, Tokyo, Japan) with a camera (SPOT™ idea, SPOT Imaging, Sterling Heights, MI, USA) and were analyzed using the corresponding software (SPOT imaging software, Sterling Heights, MI, USA) (*n* = 6).

### 2.9. Alkaline Phosphatase Assays

MG-63 cells of Passage 18 were cultured following the cell viability assay protocol for Days 1, 3, and 5. Once they reach the timepoints, cells in the wells were washed twice with phosphate-buffered saline (PBS). PBS was suctioned and 300 μL of Triton-100 at 0.05% was added. Cells went through 3 cycles, each one of 5 min at 37 °C and −4 °C for cell rupture. Later, samples were placed into 96 wells. ALP activities were determined using p-nitrophenyl phosphate (pNPP; Sigma, St. Louis, MO, USA) as the substrate. Enzyme activities were quantified from absorbance measurements at 405 nm using Multiskan™ GO Microplate Spectrophotometer (Thermo Fisher Scientific, Waltham, MA, USA) and were normalized to total protein contents, which were determined using the BCA method in aliquots of the same samples with a Pierce (Rockford, IL, USA) protein assay kit (*n* = 6).

### 2.10. Statistical Analyses

All values were expressed as mean ± standard deviation. Jarque-Bera was used to test the normality in the results. Differences between HA/β-TCP + Ar-GDP and HA/β-TCP groups were identified using Student’s *t*-tests and were considered significant when *p* < 0.05.

## 3. Results

### 3.1. SEM Observations

**Figure 2:**
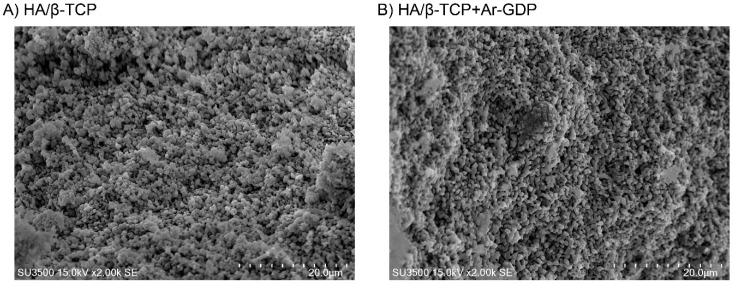
SEM images. (**A**) HA/β-TCP composite (×2000); (**B**) HA/β-TCP composite with argon glow discharge plasma treatment (×2000).

Morphological structures of HA/β-TCP + Ar-GDP and HA/β-TCP surfaces were similar. SEM analyses revealed no discernible damage associated with the surface treatment, and HA/β-TCP + Ar-GDP resembled HA/β-TCP, with rough surfaces within macro- and microporous structures that induce osteoblastic cell attachment and allow for the passage of fluids (Figure 2). Structural characteristic groups and vibration bonds were examined by Fourier Transform Infrared Spectrum (FTIR) and are presented in the supplementary materials.

### 3.2. EDS Analysis

**Figure 3:**
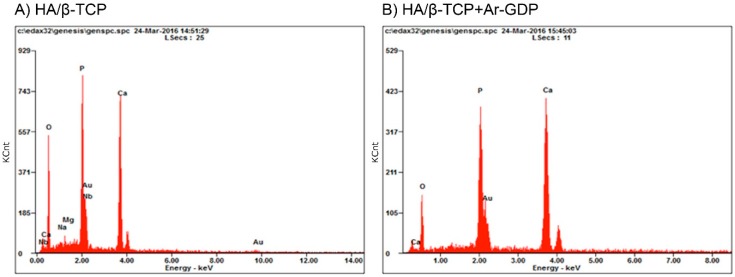
EDS spectra. The calcium and phosphate were observed on the surfaces of HA/β-TCP particles before (**A**) and after the glow discharge plasma treatment (**B**). However, the trace element impurities (Nb, Mg, and Na) were not observed after the glow discharge plasma treatment.

EDS analyses showed greater numbers of elements in HA/β-TCP than in HA/β-TCP + Ar-GDP. Specifically, magnesium, potassium, and niobium, which sometimes exist as residual impurities on HA/β-TCP surfaces, were not present after GDP treatment on HA/β-TCP + Ar-GDP. Moreover, the Ca/P mole ratio of HA/β-TCP particles was 1.34, which is not close to the stoichiometric value of HAP (1.67), while that of HA/β-TCP + Ar-GDP was 1.58, which is closer to the stoichiometric value for HA (Table 1, Figure 3).

### 3.3. XPS

**Figure 4:**
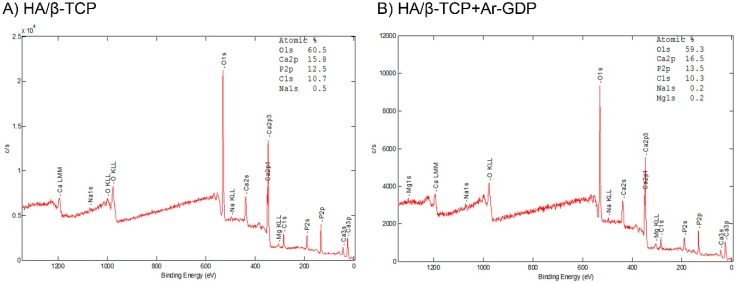
X-ray photoelectron spectroscopic analyses of atomic compositions (%). (**A**) HA/β-TCP composite (×2000); (**B**) HA/β-TCP composite with argon glow discharge plasma treatment. XPS results indicated that surface chemical bonding structure of calcium and phosphate had not changed after glow discharge plasma treatment.

XPS determinations of the atomic surface concentrations of both materials are presented in Table 2, where similar elemental composition can be seen for both materials. However, the surface atomic composition percentage showed differences after GDP treatment, meaning surface modification had occurred after the argon glow discharge treatment. (Figure 4).

**Figure 5:**
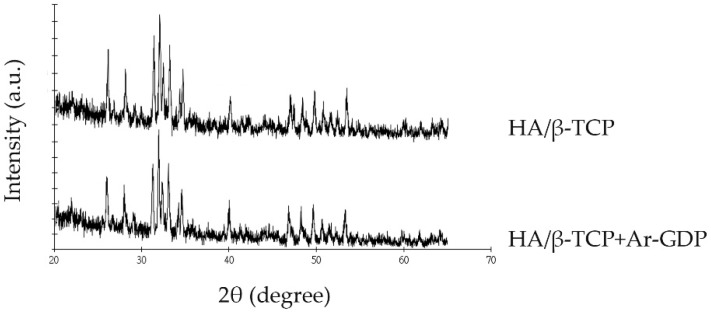
XRD patterns.

XRD patterns showed no change in the pattern as a result of the Ar-GDP. Treated and non-treated bone substitutes had the highest intensity (600 a.u.) at 27°, and this value is similar to that for HA at 21°. At 25° and 40°, HA/β-TCP + Ar-GDP and HA/β-TCP had peaks of 200 a.u. (Figure 5).

### 3.4. Cell Viability and Morphology Assessments

**Figure 6:**
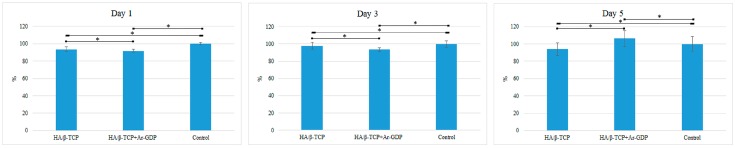
MTT assays on Days 1, 3, and 5. * Statistically significant difference (*p* < 0.05).

In MTT assays, both materials promoted osteoblastic cell proliferation, and confluent monolayers were present in all test wells, with similar pH values to those in controls. Cell viability on HA/β-TCP + Ar-GDP was 91.83% ± 1.58%, 93.46% ± 2.3%, and 106.43% ± 9.1% on Days 1, 3, and 5, respectively. In contrast, cell viability on HA/β-TCP surfaces were 93.58% ± 2.73%, 97.96% ± 3.82%, and 93.82% ± 7.1% on Days 1, 3, and 5, respectively (statistically significant difference *p* < 0.05, presented in Figure 6). MG-63 cells progressed from attachment to spreading on HA/β-TCP + Ar-GDP surfaces, leading to enhanced cell viability in comparison with that on HA/β-TCP surfaces on Day 5 (Figure 6).

### 3.5. Cell Morphology

**Figure 7:**
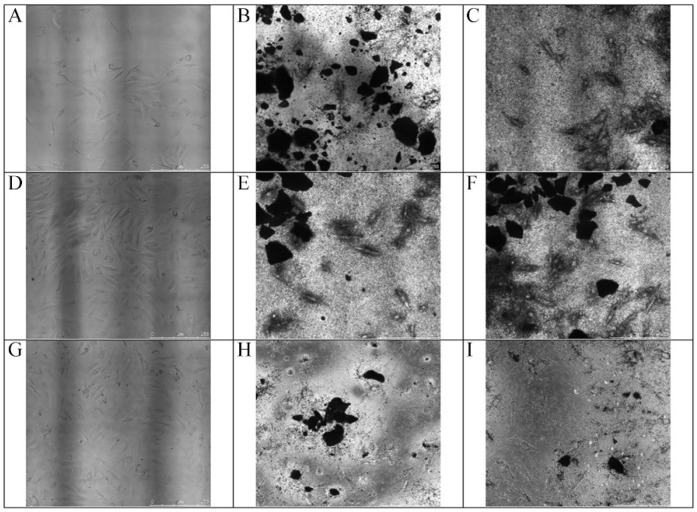
MG-63 cells co-cultured with particle grafts. Changes in MG-63 cell morphology on Days 1, 3, and 5. Magnification 40×. (**A**) Cells growing in Dulbecco’s modified Eagle’s medium on Day 1. (**B**) Cells growing with HA/β-TCP + Ar-GDP on Day 1. (**C**) Cells were cultured with HA/β-TCP and were grown for 1 day. (**D**) Cells in Dulbecco’s modified Eagle’s medium on Day 3. (**E**) Cells growing around HA/β-TCP + Ar-GDP on Day 3. (**F**) Cells growing around HA/β-TCP on Day 3. (**G**) Cells in Dulbecco’s modified Eagle’s medium on Day 5 showing a stage of development. (**H**) Cells with HA/β-TCP + Ar-GDP showing intimate contact with particle grafts on Day 5. (**I**) Cells on HA/β-TCP showed development and intimate contact with particle grafts on Day 5.

Morphology analyses of cells showed growth and spreading on both surfaces, with elongated appearances and the formation of relatively thin continuous monolayers. In the presence of biomaterials, cells surrounding the graft particles were observed on Days 1, 3, and 5 (Figure 7).

### 3.6. Alkaline Phosphatase Assay

**Figure 8:**
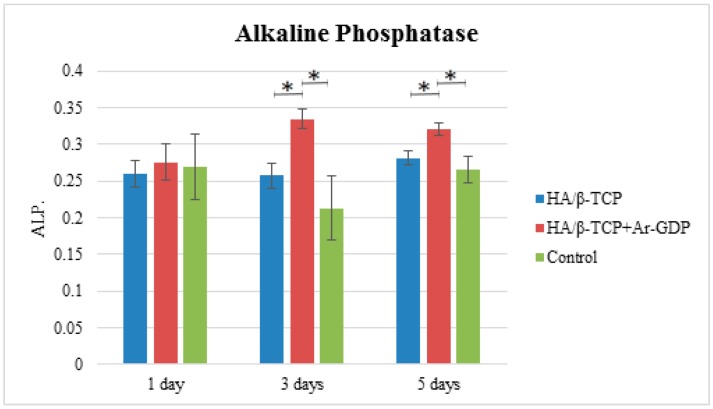
ALP assays. * Statistically significant difference (*p* < 0.05)*.*

Alkaline phosphatase tests showed that HA/β-TCP + Ar-GDP increased ALP activity in a time-dependent manner, with significantly greater enzyme activity compared to HA/β-TCP on Days 1, 3, and 5 (statistically significant difference *p* < 0.05, presented in Figure 8).

## 4. Discussion

The use of GDP as a technology for enhanced bone fusion and improved healing after skeletal injury has been investigated previously. Moreover, plasma treatments were previously used to hydrophilize surfaces of composite scaffolds and dental implants and facilitate cell adhesion [27,28,29]. Numerous studies show that the surface characteristics of biomaterials can directly influence cellular responses, thus affecting rates of growth and qualities of new tissue [30,31]. In the present study, we used Ar-GDP surface sputtering to remove element impurities from HA/β-TCP surfaces and enhance their bone regeneration properties. SEM analyses showed similar topographies of HA/β-TCP and HA/β-TCP + Ar-GDP surfaces, with rough surfaces within macro- and microporous structures that are ideal for bone regeneration and angiogenesis. The formation of a new vascular network, as Hing et al. explained, is strongly influenced by the degree of structural interconnectivity between pores, indicating that both micro- and macroporosity play a role in bone regeneration [32].

In addition, EDS analyses in the present study indicated HA/β-TCP Ca/P mole ratios lower than those previously reported of bone apatite crystals, which is due to the use of biphasic powders, where Ca/P mole ratios lower than 1.50 are easily obtained [33]. Apatite is considered calcium-deficient when the Ca/P mole ratios are lower than the stoichiometric value of 1.67 for pure calcium hydroxyapatite [34]. Though the Ca/P mole ratio of HA/β-TCP and HA/β-TCP + Ar-GDP in the present study was lower than the median reference value reported by Tzaphlidou et al. [35] in human rib bones (2.19), HA/β-TCP and HA/β-TCP + Ar-GDP allowed for cell attachment, proliferation, and expression. EDS analyses showed that HA/β-TCP + Ar-GDP had reduced contaminant concentrations. The use of XPS for surface characterization has shown this to be a technique that can provide all the information needed to distinguish the bulk composition from that of the outermost layers in particle grafts [36]. The influence of surface impurities on bioceramics is an important topic. As Franca et al. indicated in 2014, it is important to eliminate such impurities from bioceramics because they introduce critical defects, affecting their mechanical properties during lengthy implantation for bone regeneration [37].

In concordance with EDS and XPS analysis, MTT, morphology, and ALP assays, after 5 days, demonstrated superior properties for HA/β-TCP + Ar-GDP in contrast to those of HA/β-TCP, with improved cell proliferation, increased ALP activity, and enhanced differentiation into osteoblast like cells. One can attribute these differences to the HA/β-TCP plasma surface sputtering, which removed the element impurities from the surface, turning the biomaterial into a more advanced material that induced MG-63 proliferation and osteoblastic differentiation. These results are in agreement with our previous studies, in which Ar-GDP treatment combined with fibronectin grafting favored MG-63 cell adhesion, migration, and proliferation on titanium surfaces, suggesting that Ar-GDP treatment improves surface properties [37]. In addition, Mwale et al. [30] modified polymer substrate surfaces using glow discharge treatment and assessed the sensitivity of mesenchymal stem cells to subtle differences in surface chemistry. Their findings revealed that surface modifications regulate osteogenesis and modified mesenchymal stem cell differentiation.

The most common synthetic alloplastic biomaterials in current use are calcium phosphate-based (Ca-P) bioceramics [38], and these have been extensively investigated because their mineral chemistry resembles that of human bone. The present HA/β-TCP has been used successfully for guided bone regeneration in multiple dental and orthopedics treatments [39,40,41] and has been shown to safely and efficiently support dental implants [42]. These outcomes could be improved in HA/β-TCP with Ar-GDP surface treatments that decrease total surface contaminant contents and prepare stoichiometric HA/β-TCP surfaces in a highly controlled manner. Moreover, Ar-GDP sputtering on HA/β-TCP can be used without modifying the chemical composition of entire particles, as indicated by the absence of differences between HA/β-TCP + Ar-GDP and HA/β-TCP in surface characterizations and XPS analyses.

To confirm the results in the present study, in vivo testing in suitable animal models will be important for assessing HA/β-TCP + Ar-GDP biocompatibility, wound interaction, and efficacy in bone regeneration.

## 5. Conclusions

Within the limitations of this in vitro study, the present data show that GDP surface treatment only modifies HA/β-TCP chemical surfaces by eliminating contaminants and improving Ca/P mole ratios to values that more closely resemble human bone. Taken together, these experiments warrant further consideration of HA/β-TCP + Ar-GDP as a technology that improves new bone regeneration by enhancing cell attachment, proliferation, and differentiation into osteoblast like cells.

## Figures and Tables

**Table 1:** EDS analyses of particles with and without argon glow discharge plasma (Ar-GDP) treatment.

Materials	HA/β-TCP		HA/β-TCP + Ar-GDP	
Element	Weight %	Atomic %	Weight %	Atomic %
O	33.81	58.15	25.02	48.13
P	18.19	16.16	19.19	19.07
Au	13.34	1.86	16.43	2.57
Ca	31.64	21.72	39.36	30.23
Mg	0.66	0.75	0	0
Nb	1.62	0.48	0	0
Na	0.73	0.87	0	0
Total	100	100	100	100

**Table 2:** XPS analyses of atomic compositions (%).

Materials	C1s	O1s	Na1s	P2p	Ca2p
HA/β-TCP	10.72	60.5	0.46	12.53	15.79
HA/β-TCP + Ar-GDP	12.06	59.79	0.2	12.06	16.09

## Supplementary Materials

The following are available online at www.mdpi.com/1996-1944/10/12/1347/s1. Figure S1: Fourier Transform Infrared Spectrum (FTIR) Analysis. (A) HA/β-TCP particles before and (B) HA/β-TCP particles after Ar-GDP treatment. These show no change in the spectra as a result of the Ar-GDP treatment.
