# Supplementary material for: Enhancement of Osteoblastic-Like Cell Activity by Glow Discharge Plasma Surface Modified Hydroxyapatite/β-Tricalcium Phosphate Bone Substitute
Source: Materials (Basel). 2017 Nov 23;10(12):1347. doi: 10.3390/ma10121347 (PMC5744282; doi:10.3390/ma10121347)
Supplement: Supplementary file 1 [file materials-10-01347-s001.pdf]

materials

MDPI

Supplementary Information

Enhancement of Osteoblastic-Like Cell Activity by Glow Discharge Plasma Surface Modified Hydroxyapatite/β-Tricalcium Phosphate Bone Substitute

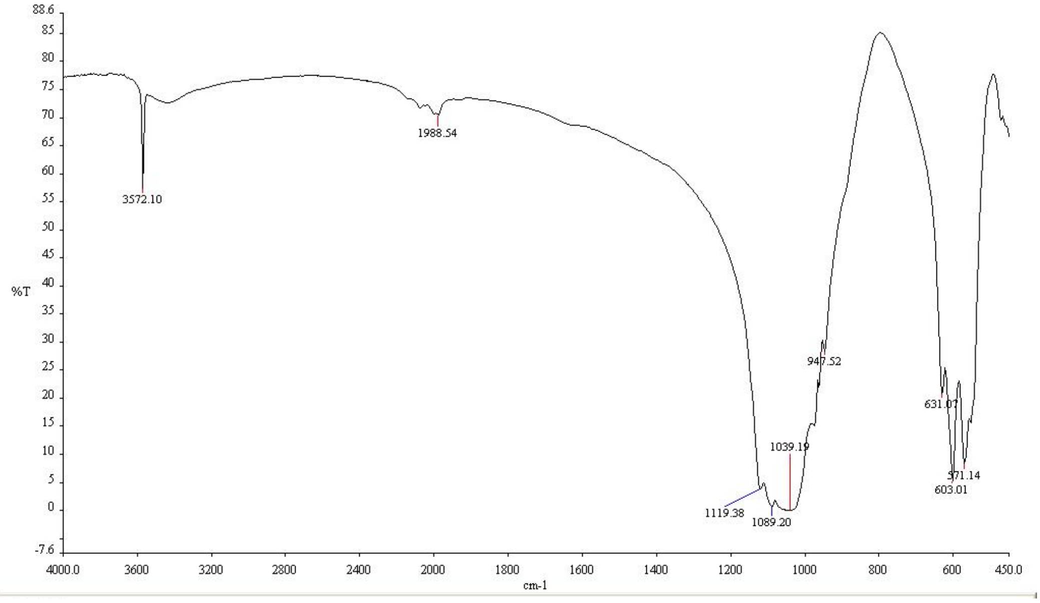

(A)

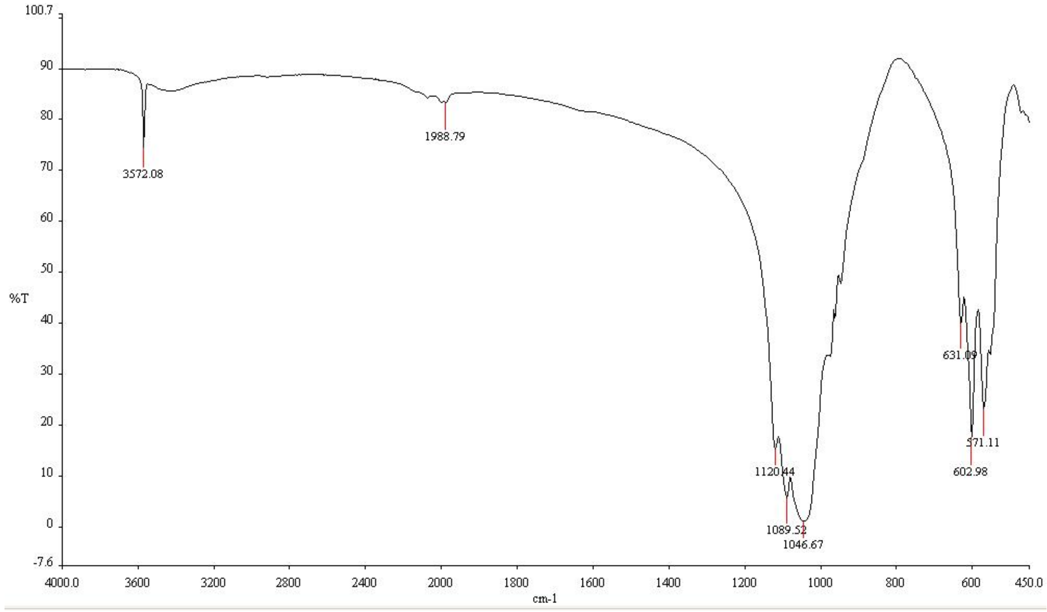

(B)

Figure S1: Fourier Transform Infrared Spectra (FTIR) for (A) HA/β-TCP and (B) HA/β-TCP+Ar-GDP.

In the IR spectra, peaks at 3570 cm^{-1}, 1040cm^{-1}, and 570–601cm^{-1} can be assigned to

stretching vibrations involving OH-, PO_4^{3-}, and CO_3^{2-} moieties in the HAP lattice. There were no significant difference in peaks between (A) HA/β-TCP and (B) HA/β-TCP+Ar-GDP.

Reference

1 Liga Berzina-Cimdina and Natalija Borodajenko. Research of Calcium Phosphates Using Fourier Transform Infrared Spectroscopy, Infrared Spectroscopy - Materials Science, Engineering and Technology. Prof. Theophanides Theophile Ed., InTech, Shanghai, China, 2012, Chapter 6.
